# Karyotype of three *Lonchophylla* species (Chiroptera, Phyllostomidae) from Southeastern Brazil

**DOI:** 10.3897/CompCytogen.v10i1.6646

**Published:** 2016-01-22

**Authors:** Brunna Almeida, Roberto Leonan Morim Novaes, Marcia Aguieiras, Renan de França Souza, Carlos Eduardo Lustosa Esbérard, Lena Geise

**Affiliations:** 1 Universidade do Estado do Rio de Janeiro, Departamento de Zoologia, Rua São Francisco Xavier 524, 20550-013, Maracanã, Rio de Janeiro, RJ, Brazil; 2 Museu Nacional do Rio de Janeiro, Departamento de Vertebrados, Quinta da Boa Vista s/n, 20940-040, São Cristóvão, Rio de Janeiro, RJ, Brazil; 3 Fundação Oswaldo Cruz, Campus Fiocruz da Mata Atlântica, Estrada Rodrigues Caldas 3400, 22713375, Taquara, Rio de Janeiro, RJ, Brazil; 4 Universidade do Estado do Rio de Janeiro, Departamento de Ecologia, Rua São Francisco Xavier 524, 20550-013, Maracanã, Rio de Janeiro, RJ, Brazil; 5 Universidade Federal Rural do Rio de Janeiro, Instituto de Biologia, Km 47 da antiga estrada Rio-São Paulo, 23851-970, Seropédica, RJ, Brazil

**Keywords:** Karyology, chromosomes, Bokermann’s Nectar Bat, Atlantic Forest, Cerrado, Endangered species, Lonchophyllinae, range extension

## Abstract

*Lonchophylla* Thomas, 1903 is a Neotropical bat genus that comprises 12 species, with little cytogenetic information available. Here we present the description of the karyotype of three species collected in Southeastern Brazil. *Lonchophylla bokermanni* Sazima, Vizotto & Taddei, 1978, *Lonchophylla dekeyseri* Taddei, Vizotto & Sazima, 1983, and *Lonchophylla peracchii* Dias, Moratelli & Esberard, 2013 showed the same diploid number 2n = 28 and the same autosomal fundamental number FNa = 50, in both *Lonchophylla bokermanni* and *Lonchophylla peracchii*. We observed that the karyotypes were also cytogenetically similar when we compared the studied species with other species within the same genus. It is therefore not possible to differentiate the species using only karyotypes with conventional staining. However, this information increases the knowledge of the genus and can be one more important character for a better phylogenetic comprehension of this taxon.

## Introduction

In recent years, new species and a genus of the subfamily Lonchophyllinae were described: *Lonchophylla peracchii* Dias, Moratelli & Esberard, 2013, *Lonchophylla inexpectata* Moratelli & Dias, 2015, and *Hsunycteris* Parlos, Timm, Swier, Zeballos & Baker, 2014 (Dias et al. 2013, Parlos et al. 2014, Moratelli and Dias 2015). For the description of bat species, morphological and morphometric characteristics are usually employed, but the use of other tools such as cytogenetic analysis can provide essential information for evolutionary relationships of bats (Varella-Garcia and Taddei 1989, Garcia and Pêssoa 2010), as already seen for rodents, for example (Romanenko and Volobouev 2012). Although there are few cytogenetic data for Lonchophyllinae, they were nevertheless informative for systematic rearrangements of this taxon (see Parlos et al. 2014).

In Brazil, there are records for five species of this genus: *Lonchophylla bokermanni* Sazima, Vizotto & Taddei, 1978, *Lonchophylla dekeyseri* Taddei, Vizotto & Sazima, 1983, *Lonchophylla inexpectata*, *Lonchophylla mordax* Thomas, 1903 and the new species, *Lonchophylla peracchii* mentioned above. There are karyotype data available until now for the two congeneric taxa from outside the country, *Lonchophylla robusta* Miller, 1912 and *Lonchophylla concava* Goldman, 1914 (Parlos et al. 2014), but no cytogenetic data were available for Brazilian species. Therefore, this study is the first to describe the karyotype of *Lonchophylla bokermanni*, *Lonchophylla dekeyseri* and *Lonchophylla peracchii*.

## Material and methods

Five individuals of *Lonchophylla* were collected and four were karyotyped: one adult female (MN79997) and one adult male of *Lonchophylla bokermanni* (MN81467), one adult female of *Lonchophylla dekeyseri* (MN80002) and one adult male of *Lonchophylla peracchii* (MN81468).

**Figure 1.**
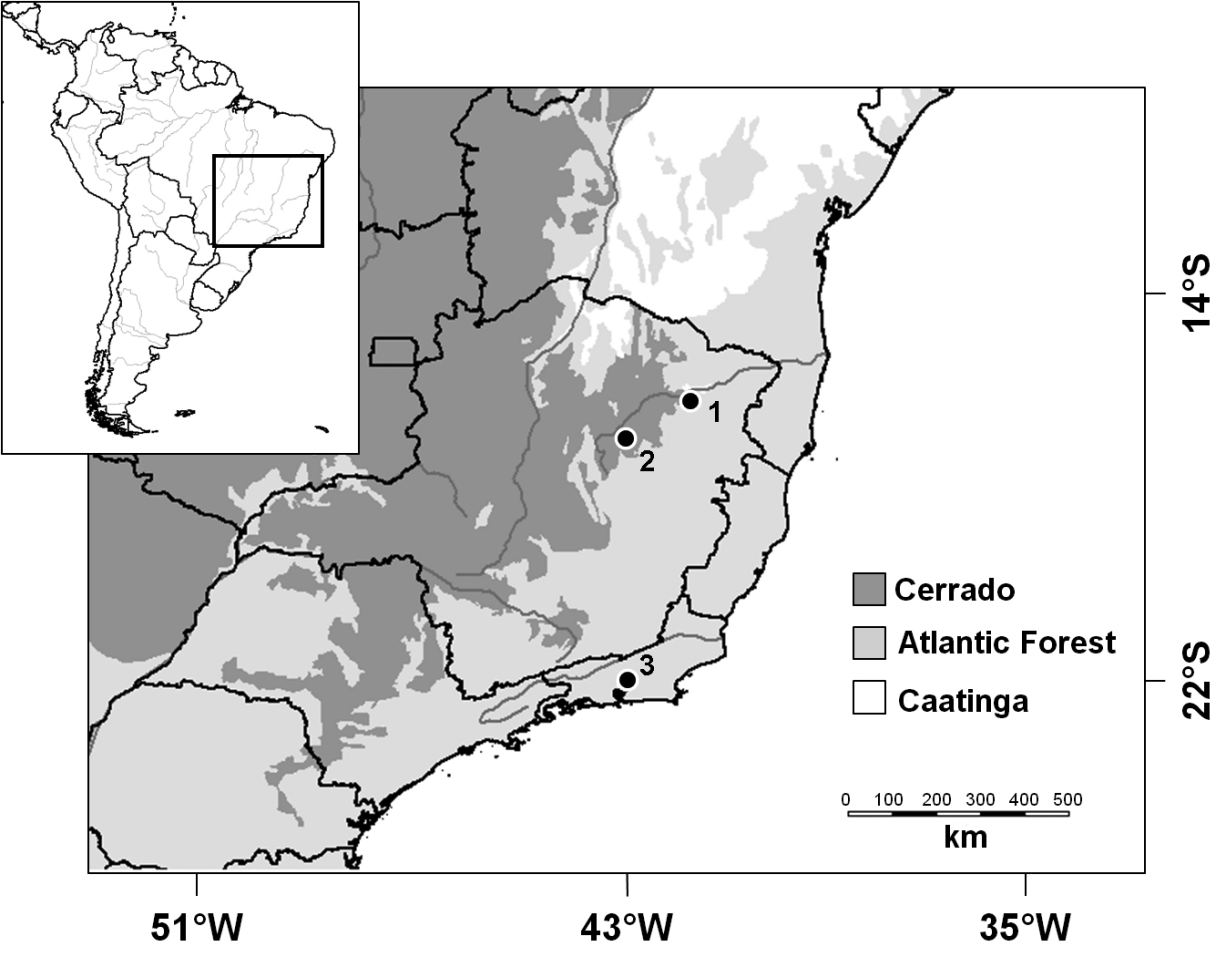
Localities of *Lonchophylla* species records: **1** Itinga, Minas Gerais **2** Dimantina, Minas Gerais **3** Magé, Rio de Janeiro.

*Lonchophylla bokermanni* was captured in Fazenda Santa Cruz, Diamantina municipality (18°16'11"S; 43°23'04"W, 1.129 m a.s.l), in the Vale do Jequitinhonha, Minas Gerais State (Figure 1). The locality has a Cerrado vegetation classified as arboreal savanna with enclaves of deciduous forest (IBGE 2012). Sampling occurred in March 2011 using 13 to 15 mist-net (9 × 3 m, 35 mm mesh), which remained open in the first six hours after the sunset for six consecutive nights.

*Lonchophylla dekeyseri* was captured in Fazenda Ilha, Itinga municipality (16°38'05"S; 41°50'54"W, 240 m a.s.l), Vale do Jequitinhonha, Minas Gerais State (Figure 1). The locality is in the Cerrado, with vegetation classified as open savanna in transition with Dry Forest (IBGE 2012). The sampling procedures were performed in March 2012, using 12 to 16 mist-nets (9 × 3 m, 35 mm mesh) that remained open in the first six hours after the sunset for seven consecutive nights.

*Lonchophylla peracchii* was captured in Reserva Particular do Patrimônio Natural El Nagual, Magé municipality (22°32'55"S; 43°03'20"W, 197 m a.s.l), Rio de Janeiro State (Figure 1). The locality is in the Atlantic Forest, with vegetation classified as Ombrophilous Dense Forest (IBGE 2012). Sampling occurred in August 2012 using two mist-nets (12 × 3 m, 30 mm mesh) that remained open throughout night period (± 12 hours) for two consecutive nights.

Chromosomes in metaphases were obtained through *in vitro* bone marrow culture grown in Dulbecco´s MEM with 10% fetal bovine serum and colchicine for 2 hours, following by an incubation in KCl 0.075M solution at 37 °C by 30 minutes, centrifuged, fixed in Carnoy solution (methanol: acetic acid, 3:1). The fixation step was repeated three times. Preparation was done by dropping one drop by distance onto clean microscope slides and air-dried. Conventional staining with Giemsa 5% was used to observe diploid number (2n) and Fundamental Number of autosomal arms (FNa) and chromosome morphology variation. This analysis was carried out using an optic photomicroscope (Nikon Eclipse 50i), in a 1,000 increase – lenses of 100 plus 10 ocular lenses.

Captures were authorized by IBAMA (1785/89-IBAMA) and SISBIO (4156/95-46 in the Vale of Jequitinhonha and 3893-1/28717 in Magé).

## Results and discussion

**Figure 2.**
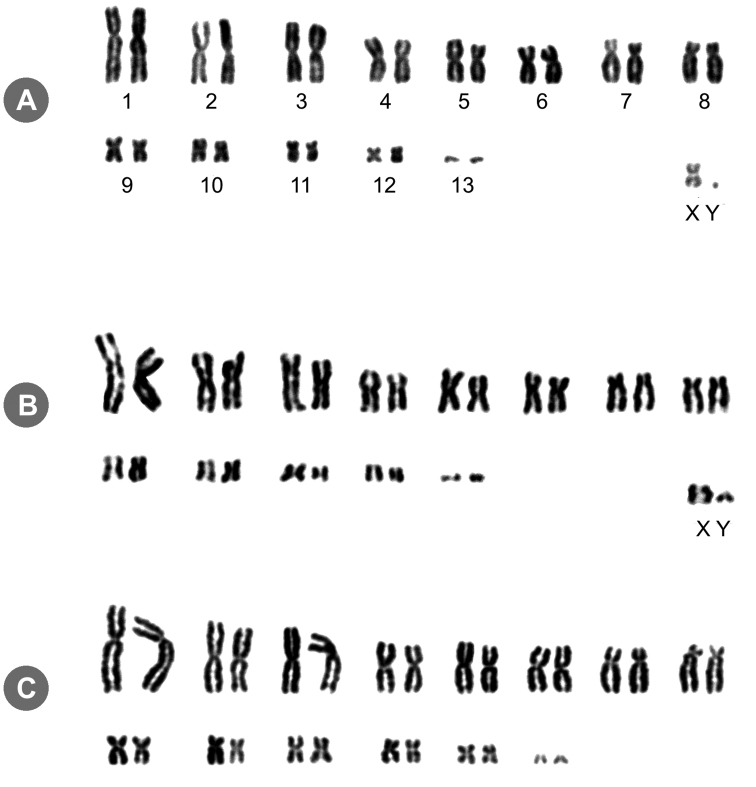
Giemsa-stained karyotypes of **A** *Lonchophylla bokermanni* 2n = 28, FNa = 50 (male, MN81467) **B** *Lonchophylla peracchii* 2n = 28, FNa = 50 (male, MN81468) and **C** *Lonchophylla dekeyseri* 2n = 28 (female, MN80002).

All three species showed the same diploid number 2n = 28 and an autosomal fundamental number FNa = 50 was observed (Figure 2). The autosomal complement of males *Lonchophylla bokermanni* and *Lonchophylla peracchii* consists of 12 pairs of meta/submetacentrics varying from large to small, and a pair of small acrocentric chromosomes (FNa = 50). Two size classes of autosomal chromosomes can be observed – the eight first are all large chromosomes, and in the second row (Figure 2A–C), with smaller ones, including four metacentric and the smallest chromosome of the karyotype, the only acrocentric ones. The X chromosome is a medium sized metacentric and the Y is a minute acrocentric, smaller than the last pair of autosomal complement. Similarly, the karyotype of *Lonchophylla dekeyseri* can be characterized but the identification of the sex chromosome pair was impossible in the sole collected specimen which was a female.

Karyotype comparison is considered as an important tool to establish phylogenetic relationships and as a taxonomic tool to confirm some species identities (Baker 1970, Silva et al. 2006, Urdampilleta et al. 2013). However, the resolution power of the cytogenetic method is not the same for all groups. Sometimes, it is necessary to analyze as many as possible the species’ karyotypes (Garcia and Pessoa 2010). In bats, the few available published karyotype data from South America (Moratelli and Morielle-Versute 2007) make it difficult to propose, using such kind of information, new or different taxonomic arrangements and a better comprehension of the systematics of Neotropical bats (Garcia and Pessoa 2010).

Three new karyotypes here described for *Lonchophylla bokermanni*, *Lonchophylla dekeyseri* and *Lonchophylla peracchii* are similar to those known for *Lonchophylla robusta* (Baker 1973, Baker 1979) and *Lonchophylla concava* (Parlos et al. 2014). A species currently allocated to the genus *Hsunycteris* and previously described as *Lonchophylla thomasi*, presents different karyotype compositions: 2n = 30, FNa = 34; 2n = 32, FNa = 34, 38 and 40; 2n = 36, FNa = 48. Additionally, this species also presented an increased number of acrocentric chromosomes, whereas in other *Lonchophylla* species, only a pair of small acrocentric is observed (Pair 13 in Figure 2) (Parlos et al. 2014).

The karyotype conservatism in Microchiroptera has been observed in other studies (Varella-Garcia et al. 1989, Sousa and Araújo 1990) which well corroborate with our results. Even if species distinction is not evident for representatives of the *Lonchophylla* genus through the conventional chromosome characteristics, the generic separation of *Lonchophylla* – *Hsunycteris* is supported by their different karyotypes.
